# Peroxiredoxin 1 – an antioxidant enzyme in cancer

**DOI:** 10.1111/jcmm.12955

**Published:** 2016-09-21

**Authors:** Chenbo Ding, Xiaobo Fan, Guoqiu Wu

**Affiliations:** ^1^ Medical School of Southeast UniversityNanjingChina; ^2^ Center of Clinical Laboratory MedicineZhongda HospitalSoutheast UniversityNanjingChina

**Keywords:** peroxiredoxin 1, reactive oxygen species, cancer

## Abstract

Peroxiredoxins (PRDXs), a ubiquitous family of redox‐regulating proteins, are reported of potential to eliminate various reactive oxygen species (ROS). As a major member of the antioxidant enzymes, PRDX1 can become easily over‐oxidized on its catalytically active cysteine induced by a variety of stimuli *in vitro* and *in vivo*. In nucleus, oligomeric PRDX1 directly associates with p53 or transcription factors such as c‐Myc, NF‐κB and AR, and thus affects their bioactivities upon gene regulation, which in turn induces or suppresses cell death. Additionally, PRDX1 in cytoplasm has anti‐apoptotic potential through direct or indirect interactions with several ROS‐dependent (redox regulation) effectors, including ASK1, p66^Shc^, GSTpi/JNK and c‐Abl kinase. PRDX1 is proven to be a versatile molecule regulating cell growth, differentiation and apoptosis. Recent studies have found that PRDX1 and/or PRDX1‐regulated ROS‐dependent signalling pathways play an important role in the progression and metastasis of human tumours, particularly in breast, oesophageal and lung cancers. In this paper, we review the structure, effector functions of PRDX1, its role in cancer and the pivotal role of ROS in anticancer treatment.

## Introduction

Reactive oxygen species (ROS), including superoxide (O_2_^−^), hydrogen peroxide (H_2_O_2_) and hydroxyl radical (OH^•^), are converted directly or indirectly from free oxygen but are more chemically reactive 1, 2. The low‐to‐moderate ROS level is indispensable to normal cellular proliferation, differentiation and survival 3. The net emission of ROS depends upon the balance between free radical production (pro‐oxidative process) and its elimination by antioxidants (antioxidant defense process) 4. Peroxiredoxins (PRDXs), a family of this kind of antioxidants, are classified on the basis of having either one (1‐Cys) or two (2‐Cys) conserved cysteine residues 5. PRDX1 is a member of the 2‐Cys PRDXs subfamily and is present mainly in the cytosol 6. PRDX1 containing a cysteine at the N‐terminal Cys^52^ detoxifies peroxides at the expense of Cys^52^ oxidation through intermolecular disulphide formation with the other conserved C‐terminal Cys^173^ residue 7. Oxidized PRDX1 could be converted to its active form by various mechanisms 7, 8.

PRDX1 was firstly reported as an antioxidant enzyme, but its physiological role in oxidization–reduction balance remains unclear because it is highly susceptible to oxidative stress. Upon peroxidatic Cys oxidation, 2‐Cys PRDX1 is structurally converted from a peroxidase enzyme to a molecular chaperone under stress conditions 9, 10. In addition to its peroxidase and chaperone functions, PRDX1 could also enhance natural killer cell cytotoxicity and suppress oncogenic proteins such as c‐Myc and c‐Abl 11, 12, 13. Recent studies have shown that abnormal expression of PRDX1 has been observed in several human cancers, including breast, oesophageal, lung and prostate cancers 14, 15, 16, 17. Furthermore, PRDX1 also regulates several ROS‐dependent signalling pathways and is thought to be a key intracellular intermediate balancing cell survival and apoptosis 7, 18.

In this review, we provide an overview of the structure, effector functions of PRDX1 and its role in ROS‐dependent signalling pathways. We also summarize the recent advances implicating PRDX1 in cancer development and the importance of targeting ROS‐dependent/redox pathways in anticancer treatment.

## Structure, functions of PRDX1 and its role in ROS‐dependent signalling

Mammalian PRDXs are divided into three categories based on their number of conserved Cys residues and catalytic mechanism: 2‐Cys PRDXs (PRDX1–4), atypical 2‐Cys PRDX (PRDX5) and 1‐Cys PRDX (Prx6) 5. All PRDXs contain a thioredoxin fold with a few additional secondary structure elements present as insertions 5. PRDX1 contains a conserved Cys^52^ at the N‐terminal and a conserved Cys^173^ at the C‐terminal 7. PRDX1 is domain‐swapped homodimers in which the C terminus of one subunit reaches across the dimer interface to interact with the other subunit 8. In addition, PRDX1 crystallized as toroid‐shaped complexes consisting of a pentameric arrangement of dimers [an (a_2_)_5_ decamer], consistent with observations that PRDX1 dimers can form discrete higher‐order oligomers 8. Compared with other antioxidant enzymes, PRDX1 employs a particular mechanism to detoxify peroxide with reducing equivalents provided through the thioredoxin (Trx) system but not from glutaredoxin 19.

**Figure 1:**
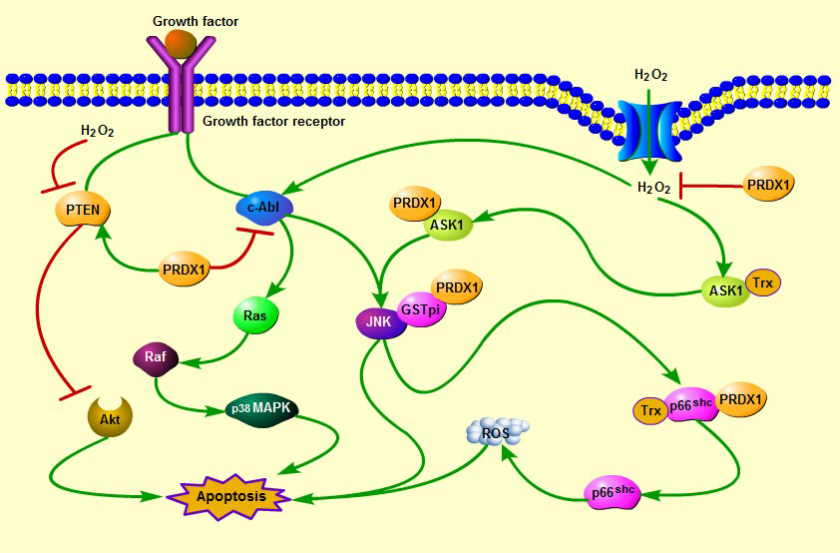
The roles of cytoplasm PRDX1 in the oxidative stress‐induced apoptosis. PRDX1 cooperates with Trx in suppressing H_2_O_2_‐induced cell death involving different types of kinases and enzymes, such as ASK1, p66^Shc^ and GSTpi/JNK. In addition, PRDX1 could inhibit JNK and p38 MAPK‐induced cell apoptosis through direct interaction with c‐Abl tyrosine kinase under oxidative stress conditions. Notably, PRDX1 prevents Akt‐driven tumourigenesis and then promotes cell apoptosis through protecting PTEN lipid phosphatase activity from oxidation‐induced inactivation.

Previous studies have indicated that PRDXs or Trx peroxidases are important endogenous antioxidants, protecting cells from oxidative damage by reducing H_2_O_2_ and peroxynitrite and scavenging thiyl radicals 20, 21, 22. PRDX1 cooperates with Trx in suppressing H_2_O_2_‐induced cell death involving different types of kinases and enzymes, such as apoptosis signal‐regulating kinase 1 (ASK1), p66^Shc^ and glutathione *S*‐transferase pi (GSTpi)/c‐Jun NH2‐terminal kinase (JNK), which play key roles in the regulation of cell death and/or apoptosis depending on cell type and stimuli 23, 24, 25. Additionally, PRDX1 is known as a physiological inhibitor of c‐Abl tyrosine kinase. It could bind the SH‐3 domain of c‐Abl 13. As c‐Abl plays a crucial role in oxidative stress‐induced cell death and is known to function as an upstream effector of the JNK and p38 mitogen‐activated protein kinase (MAPK) pathways 18, 26, we speculate that PRDX1 may play a role in suppressing stress‐induced cell death through ROS‐dependent signalling pathway (Fig. 1). Notably, PRDX1 prevents Akt‐driven tumourigenesis and then induces cell death through protecting phosphatase and tensin homologue (PTEN) lipid phosphatase activity from oxidation‐induced inactivation 7.

**Figure 2:**
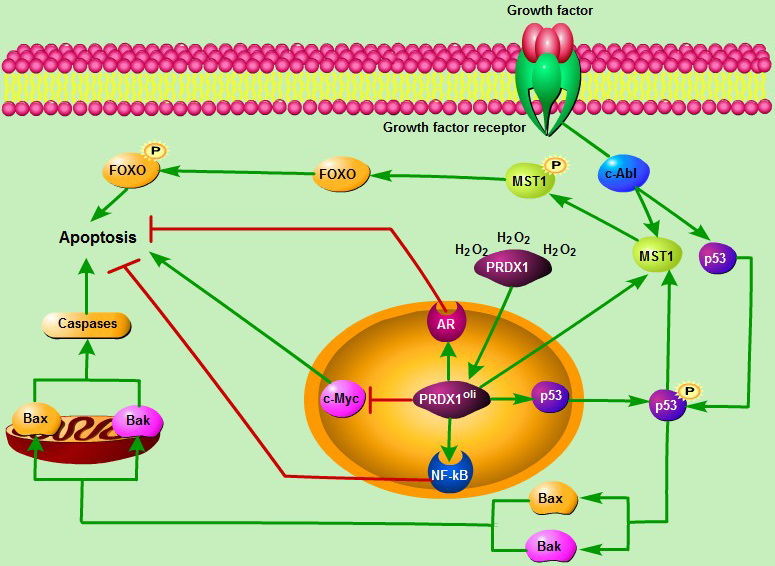
The roles of nucleus PRDX1 in the H_2_O_2_‐induced apoptosis. It is also known that PRDX1 functions as a chaperone in the form of oligomers, and molecular chaperone activities enhanced under oxidative stress conditions. PRDX1 oligomer directly interacts with the transcription factors including c‐Myc, NF‐κB and AR, and thus affects their bioactivities upon gene regulation, which in turn induces or suppresses cell death. In addition, p53 induces the expression of apoptosis factors such as Bak and Bax under oxidative stress conditions, and promotes the activation of caspases and p53‐dependent mitochondria apoptotic signalling pathway. Importantly, PRDX1 oligomer is an essential intermediate in H_2_O_2_‐induced activation of c‐Abl/MST1/FOXO signalling pathway and cell apoptosis through direct interaction with p53. Furthermore, it has been recognized that c‐Abl acts as a modulator of p53. PRDX1^oli^, PRDX1 oligomer.

The function of PRDX1 is not restricted to its antioxidant activities in cancers. It is also known that PRDX1 functions as a chaperone in the form of oligomers, and molecular chaperone activities enhanced under oxidative stress conditions 27, 28. PRDX1 oligomer could interact with the c‐Myc oncogene and suppresses its transcriptional activity, which in turn inhibits tumourigenesis and promotes tumour cell apoptosis 29. However, PRDX1 may act as an oncogene, and suppresses tumour cell death by directly associating with transcription factors such as nuclear factor kappa B (NF‐κB) and androgen receptor (AR) 30, 31. It has been widely recognized that p53‐dependent apoptosis signalling is participated in the oxidative stress‐induced cell death 32. P53 induces the expression of apoptosis factors such as Bak and Bax under oxidative stress conditions, which in turn promotes the activation of caspases, and then activates the mitochondrial apoptotic signalling pathway. Interestingly, oligomeric PRDX1 is an essential intermediate in H_2_O_2_‐induced mammalian Ste20‐like kinase‐1 (MST1) activation and cell apoptosis through p53 33. In addition, oxidative stress induces the c‐Abl‐dependent tyrosine phosphorylation of MST1 and increases the interaction between MST1 and FOXO3 (Forkhead box O3), thereby activating the MST1–FOXO signalling pathway and leading to cell death 34. It has been demonstrated that c‐Abl is also known as a modulator of p53 signalling pathway 35.These studies suggest that oligomeric PRDX1 may play a key role in modulating stress‐induced apoptosis through its interaction with p53 or nuclear transcription factors (Fig. 2).

## Genomic studies in cancer

DNA amplification or chromosomal losses is a common mechanism leading to oncogenic activation in human cancers. *PRDX1* gene is located on chromosomal band 1q34.1. Loss of heterozygosity (LOH) of 1q has been suggested in tumour progression 36, 37, 38. Mice lacking PRDX1 develop severe haemolytic anaemia and various types of malignancies, indicating that PRDX1 functions as a tumour suppressor 39. In the region of 1q33‐34, Sulman E P *et al*. have identified several tumour suppressor candidates, including *MUTYH*,* PRDX1*,* FOXD2*,* FOXE3*,* PTCH2* and *RAD54L* genes in meningioma 40. However, *PRDX1* gene mutations have not been identified in human tumours. Interestingly, several studies have confirmed that the *PRDX1* gene is overexpressed in human malignancies, suggesting that PRDX1 may be a proto‐oncogene.

The identification of PRDX1 as a potential oncogene was reported in the cases of lung adenocarcinomas 41, 42, colorectal cancer 43 and soft tissue sarcomas 44. 1q is amplified in approximately 35.5% of lung cancer 45, and the PRDX1 expression is significantly elevated in lung cancer patients as compared with patients with benign lung disease 46. Li R *et al*. found that elevated expression of PRDX1 is characterized of both increased DNA copy number and increased transcript levels in lung adenocarcinoma 41. Recurrent gains were found for chromosome 1q in colorectal cancer 47, 48. *PRDX1* was obviously amplified in colorectal primary tumours compared with normal colon samples by the significance analysis of microarrays 43. Taken together, these genomic studies validate the presence of *PRDX1* amplification in human cancers.

## PRDX1 and breast cancer

The specific role for PRDX1 in mammary carcinomas is controversial. PRDX1 protein was found to be overexpressed in breast cancer tissues from most patients compared with normal tissues, but no significant relationship was found between PRDX1 levels and clinicopathological factors, including oestrogen receptor (ER) status 14, 49. Similar to these data, Wang X *et al*. found that PRDX1 is more abundant in breast cancer cell lines (including ER+ and ER‐) than in normal or pseudonormal breast cell lines, but not found a significant difference in PRDX1 levels between ER+ and ER‐ breast cancer cell lines 50. However, other studies have shown that elevated mRNA expression of PRDX1 in human breast carcinoma is relevant with higher tumour grade 51, and high expression of cytoplasmic PRDX1 correlated with a great risk of local recurrence after radiotherapy 52. PRDX1 could act as a chaperone to enhance the transactivation potential of NF‐κB in ER‐breast cancer cells, and then suppresses tumour cell death 50. Meanwhile, PRDX1 has a protective function in doxorubicin/peroxide‐induced cytotoxicity of breast cancer cells 53, 54. Repression of PRDX1 expression leads to high levels of ROS‐induced phosphorylation of p38 MAPKα, and promotes H_2_O_2_‐induced senescence in breast cancer cells 55. It was also found that drug resistance formation is accompanied by a significant increase in the expression of *PRDX1* gene in breast cancer cell strains, which confirms the important contribution of redox‐dependent mechanisms to the development of cisplatin resistance of cancer cells 56.

Conversely, there are several reports indicated that PRDX1 may act as a tumour suppressor in breast cancer. *PRDX1*‐deficient mice suffer from shortened lifespan owing to the development of haemolytic anaemia and several malignant cancers including breast carcinomas 39. Simultaneous expression of PRDX1 and c‐Myc in *PRDX1*‐deficient mice cells apparently attenuates regulation of some c‐Myc target genes and thus inhibits tumour growth, indicating a role for PRDX1 as a tumour suppressor 12, 29. In addition, PRDX1 oligomer mediates cisplatin‐induced MST1 activation and p53‐dependent cell death in breast cancer 33. It has been reported that high PRDX1 expression appears to be associated with less aggressive breast tumours 57. In addition, Cao J *et al*. have identified that PRDX1 protects the tumour suppressive function of PTEN phosphatase from ROS‐induced inactivation, and inhibits Ras‐driven mammary tumours 58. Notably, biomarker studies have confirmed that PRDX1 is one of the diagnostic biomarkers for invasive ductal carcinoma of breast with human epidermal growth factor receptor‐2 (HER2)‐enriched subtypes 59, and PRDX1 protects ERα from oxidative stress‐induced suppression and is a protein biomarker of favourable prognosis in mammary tumours 60.

miRNAs post‐transcriptionally repress gene expression mostly by binding to the 3′UTR of mRNA transcripts to either cause degradation or prevent translation, depending upon complementarity. It was identified that miR‐510 is elevated in breast tumour samples while absent in the matched non‐tumour breast tissue samples 61. Overexpression of miR‐510 leads to decreased PRDX1, which, in turn, increases the activity of PI3K/Akt pathway and promoted cell and tumour growth in breast cancer 62. This exemplifies yet another mechanism of PRDX1 regulation in breast carcinogenesis.

## PRDX1 and oesophageal cancer

Similar to breast cancer, the functions of PRDX1 in oesophageal cancer remain enigmatic. Proteomic study has found that the expression level of PRDX1 is elevated in oesophagus squamous cell carcinoma (ESCC) tissue 63. Further study demonstrated that PRDX1 is significantly increased in ESCC tissues compared with the paired adjacent normal tissues 15. In addition, several studies have identified that PRDX1 is overexpressed in ESCC cells compared with the non‐cancerous oesophageal epithelial cells 64, 65. Elevated PRDX1 promotes tumourigenesis through regulating the activity of mTORp70S6K pathway in ESCC 65. Interestingly, silencing PRDX1 leads to increased levels of tumour suppressor genes LKB1 and p‐AMPK, and decreases the oncogene Aurora A expression, suggesting that PRDX1 may affect carcinogenesis 64. Apart from these, PRDX1 also has a protective function in dioscin/ionizing radiation‐induced ROS accumulation in oesophageal cancer cells 66, 67. These data highlight that PRDX1 promotes tumourigenesis by functioning as “accomplices” of certain oncoproteins or by the activity of its antioxidant enzyme.

However, several lines of evidence suggest that PRDX1 may play a role as a tumour suppressor in oesophageal cancer. p21^WAF1^, a cyclin‐dependent kinase inhibitor, has been reported to play an important role in the maintenance of cell cycle arrest 68, 69, 70. Histone deacetylase inhibitor FK228 induces growth inhibition and apoptosis in human ESCC cells, at least partially through activating the *PRDX1* gene with histones H3 and H4 acetylation of its promoter, resulting in elevated p21^WAF1^ expression 71. Accordingly, Hoshino I *et al*. showed that high PRDX1 expression appears to be associated with longer overall survival in ESCC 72.

## PRDX1 and lung cancer

Previous studies have shown that PRDX1 is expressed at significantly higher levels in lung cancer tissues compared with normal lung tissues 73, 74, 75, 76, and elevated PRDX1 associated with shorter survival in non‐small cell lung cancer (NSCLC) 77, 78. Furthermore, PRDX1 is up‐regulated in NSCLC tissue interstitial fluid, and high level of PRDX1 expression is related with lymph node metastasis and tumour differentiation, suggesting that PRDX1 may act as a marker of neoplastic progression 46. Knockdown of PRDX1 in lung cancer cells significantly inhibits transforming growth factor β1 (TGF‐β1)‐induced epithelial–mesenchymal transition (EMT) and cell migration, whereas PRDX1 overexpression enhances TGF‐β1‐induced EMT and cell migration 79. In addition, *in vivo* studies have shown that silencing of PRDX1 leads to tumour suppression of growth and metastases through reducing the activation of c‐Jun 80, 81. However, a recent study suggests that PRDX1 functions as a nuclear erythroid 2‐related factor 2 (Nrf2) dependently inducible tumour suppressant in *K‐ras*‐driven lung tumourigenesis by prohibiting ROS/ERK/cyclin D1 pathway activation 82.

PRDX1 also suppresses drug/radiation‐induced cytotoxicity in lung cancer, and the related mechanisms are under investigation 25, 83. PRDX1 knockdown evokes an increase in cellular apoptotic potential through activation of the caspase cascade and suppression docetaxel‐induced phosphorylation of Akt and its substrate FOXO1 in A549 xenograft tumours 83, 84. In addition, PRDX1 suppresses JNK activation through an indirect interaction with JNK in a peroxidase‐independent manner 25. GSTpi has been shown to play a great role in suppressing JNK activation by forming the GSTpi‐JNK complex 85, 86, 87. When cells are exposed to ionizing radiation or ROS, GSTpi is oxidized, thereby triggering a dissociation of the GSTpi‐JNK complex and releasing JNK. Interestingly, PRDX1 physically interacts with GSTpi and suppresses JNK release/activation from the GSTpi‐JNK complex after γ‐ray radiation exposure in human lung cancer 1170i cells 25.

As mentioned above, PRDX1 is a potential target for cancer radiotherapy and/or chemotherapy. Several studies have suggested that anticancer molecules/gene reverse radioresistance through targeting PRDX1 on human lung cancer 88, 89, 90. Vitamin K3 (vitK3) is a synthetic naphthoquinone exhibiting significant anticancer activity against multiple human cancers both *in vitro* and *in vivo* 91, 92, 93, and its major anticancer mechanism is depended on the generation of ROS 94, 95. Increased ROS accumulation inhibits PRDX1 expression and induces lung cancer cell death after vitK3 treatment 96. In addition, after the treatment of JS‐K, a nitric oxide prodrug, oxidative/nitrosative stress is evoked in lung adenocarcinoma cells with high basal levels of ROS/reactive nitrogen species (RNS), and PRDX1 correlating with drug dose 97. PRDX1 is a useful biomarker for identifying tumours response to therapy as PRDX1 protein can be easily measured in lung cancer 98, 99.

## PRDX1 and prostate cancer

PRDX1 overexpression is also found in human prostate cancer specimens and prostate cancer cell lines 17, 100. Prostate cancer risk and prognosis are adversely associated with a number of inflammatory and angiogenic mediators, including Toll‐like receptor 4 (TLR4), NF‐κB and VEGF 101, 102, 103, 104. Elevated PRDX1 increases prostate tumour vasculature, and shows up‐regulation of angiogenic proteins such as VEGF in the tumour region. Conversely, silencing of PRDX1 in prostate cancer cell lines reduces tumour vascular formation, and causes down‐regulation of VEGF 17, 105. PRDX1 mediates these effects by activated TLR4 and NF‐κB, which results in the increased expression of hypoxia inducible factor‐1α (HIF‐1α), a transcription factor involved in angiogenesis 105. This novel PRDX1‐TLR4‐ HIF‐1α pathway can be targeted by TLR4 inhibitors and can perhaps serve as a therapeutic target in the treatment of PRDX1‐driven prostate tumour angiogenesis.

In addition, AR signalling is integral to the development and progression of prostate cancer 106. Hypoxia/reoxygenation activates the AR in cultured prostate cancer cells, and PRDX1 acts as a key mediator as monitored with an AR‐dependent luciferase reporter assay 107. PRDX1 overexpression enhances the AR activity in response to hypoxia/reoxygenation, whereas PRDX1 knockdown has the opposite effect and reduces the growth rate of the androgen‐dependent cancer cells 107. Interestingly, the AR stimulatory function of PRDX1 is independent of its antioxidant activity 31, 107. PRDX1 enhances AR function in prostate cancer cells through increasing affinity of AR to dihydrotestosterone (DHT) 31. In PRDX1‐rich LNCap cells, the combination of PRDX1 knockdown and finasteride, a structural analogue of DHT, was found to produce a greater inhibitory effect on AR activity and cell growth than either treatment alone 108. The above findings suggest that PRDX1 may play a critical role in the context of developing strategies to improve the outcome of androgen deprivation therapy in prostate cancer. Furthermore, a train of evidence indicates that PRDX1 and other ROS‐regulated enzymes are targets for modulating intracellular redox status in therapeutic strategies for prostate cancer 109, 110, 111.

## PRDX1 and other types of malignancy

The levels of PRDX1 expression are significantly increased in pancreatic cancer compared to normal tissues, and this overexpression is closely related to tumour angiogenesis 112. In addition, PRDX1 associates with the formation of membrane protrusions through modulation of the activity of p38MAPK, which in turn promotes pancreatic cancer cell invasion 113. Although the expression of PRDX1 is weak in cervical cancer 114, PRDX1 knockdown significantly enhances HeLa cell sensitivity to ROS‐generating drugs 115. A previous study has reported that PRDX1 suppresses proteasome inhibitor‐mediated cell death through the influence on ASK1 activation in human thyroid cancer 116. However, Nicolussi A *et al*. have demonstrated that PRDX1 and PRDX6 are reduced in papillary thyroid carcinomas by BRAF V600E‐dependent and ‐independent mechanisms 117. Additionally, down‐regulation of PRDX1 suppresses growth and promotes apoptosis in bladder cancer cells through NF‐kB signalling pathway after Bifidobacterium infantis thymidine kinase/nucleoside analogue ganciclovir (BI‐TK/GCV) treatment 118. Overexpression of PRDX1 has also been observed in gallbladder cancer 119, cholangiocarcinoma 120, 121, liver cancer 122 and abdominal aortic aneurysm 123. Recently, reduced expression of PRDX1 is frequent in oligodendroglial tumours with 1p/19q deletion and likely contributes to radio/chemosensitivity of these tumours 124, 125. Subsequently, the decreased expression of PRDX1 and PRDX2 has also been identified in melanomas 126, and PRDX2 represses melanoma metastasis by increasing E‐cadherin/β‐catenin complexes in the plasma membrane 127. Considering that PRDX1 and PRDX2 belong to typical 2‐Cys PRDXs, and have similar active sites 8, we therefore hypothesize that PRDX1 may also play important roles in the suppression of melanoma development. Interestingly, the expression and role of PRDX1 in B cell‐derived malignancies is controversial. PRDX1 expression is high in multiple myeloma, but absent in plasmablastic lymphoma 128. However, Trzeciecka A *et al*. have demonstrated that PRDX1 and PRDX2 are up‐regulated in Burkitt lymphoma, and lymphoma cells treated with SK053 trigger the formation of covalent PRDX dimers, accumulation of intracellular reactive oxygen species, phosphorylation of ERK1/2 and AKT and attenuate the cells growth rate 129. By contrary, high total PRDXs is correlated with favourable disease‐specific survival and overall survival in patients with follicular lymphoma 130.

## Conclusion and future perspectives

As discussed above, the expression and functional roles of PRDX1 in many human tumours remain controversial (summarized in Table 1), and further researches need to be done to reveal its underlying role in the progression and metastasis of cancer. In spite of the various functions of PRDX1 in cancers, we could acutely discover that when PRDX1 acts as antioxidant enzyme but not chaperone, it plays a positive role in radio/chemoresistance. The current PRDX1‐based anticancer studies are mainly based on its antioxidant activity. Although results from preclinical trial, both *in vivo* and *in vitro*, for PRDX1‐based anticancer are promising, there is a long path from laboratory to clinical trial. Another important concern is off‐target effect. Is the drug targeting PRDX1 sufficient for controlling the final anticancer effects when other PRDXs and/or ROS‐independent tyrosine kinase are present? If the answer is positive, then what is the underlying mechanism? Only these events are fully explored, can the treatment targeting PRDX1 be used in clinical application.

**Table 1:** Expression and functional characterization of PRDX1 in cancer

Name	Expression	Role	Target gene	References
Breast cancer	Up‐regulated	Oncogene	NF‐κB	50
	Up‐regulated	Tumour suppressor	c‐Myc, p53	12, 29, 33, 39
Oesophageal cancer	Up‐regulated	Oncogene	mTOR	15, 65
	Up‐regulated	Tumour suppressor	Not mentioned	71
Lung cancer	Up‐regulated	Oncogene	GSTpi‐JNK, c‐Jun	25, 80, 81
Prostate cancer	Up‐regulated	Oncogene	AR	31, 107
Pancreatic cancer	Up‐regulated	Oncogene	p38 MAPK	112, 113
Cervical cancer	Weak	Chemoresistance	Not mentioned	114, 115
Thyroid cancer	Up‐regulated	Oncogene	ASK1	116
	Down‐regulated	Tumour suppressor	BRAF	117
Oligodendroglial tumour	Down‐regulated	Radio/chemosensitivity	Not mentioned	124, 125
Melanoma	Down‐regulated	Not mentioned	Not mentioned	126
B cell‐derived lymphoma	Up‐regulated	Oncogene	SK053	129
	Down‐regulated	Not mentioned	Not mentioned	128

Moreover, several important scientific issues remain to be addressed. The basal levels of ROS are higher in *PRDX1‐/‐* murine embryonic fibroblasts and erythrocytes, whereas no significant differences are observed in either splenocytes or hepatocytes, indicating that PRDX1 controls ROS levels in certain tissues 29. Interestingly, Demasi AP *et al*. have demonstrated that PRDX1 is expressed in plasma cells but not in B lymphocytes, suggesting that its expression is development‐associated 128. Notably, PRDX1 is highly susceptible to oxidative stress and its molecular chaperone activities enhanced under oxidative stress conditions. In addition, PRDX1 often cooperates with other PRDXs and/or redox‐regulating proteins such as Trx in suppressing H_2_O_2_‐induced cell death. According to these data, we speculate that the plausible reasons for the ambiguous expression and function of PRDX1 in tumours are listed as following: (*i*) PRDX1 expression is development‐associated in some tissues; (*ii*) Redox activity and chaperone activity, which play a dominant role decided the role of PRDX1 in promoting or suppressing oncogenesis for certain types of cancer; (iii) PRDX1 may have specificity in some tissues or populations; (iv) The final functional role of PRDX1 is influenced by the synergy or suppression of other PRDXs and/or redox‐regulating molecules.

## Conflicts of interest

No conflict of interest.

## Author contribution

Chenbo Ding was responsible for the conception and design of the manuscript. Chenbo Ding participated in drafting the manuscript. Xiaobo Fan and Guoqiu Wu were responsible for the review and/or revision of the manuscript. All authors read and approved the final manuscript.
